# Effect of Protein Binding on Exposure of Unbound and Total Mycophenolic Acid: A Population Pharmacokinetic Analysis in Chinese Adult Kidney Transplant Recipients

**DOI:** 10.3389/fphar.2020.00340

**Published:** 2020-03-20

**Authors:** Changcheng Sheng, Qun Zhao, Wanjie Niu, Xiaoyan Qiu, Ming Zhang, Zheng Jiao

**Affiliations:** ^1^Department of Pharmacy, Huashan Hospital, Fudan University, Shanghai, China; ^2^Department of Nephropathy, Huashan Hospital, Fudan University, Shanghai, China

**Keywords:** population pharmacokinetics, nonlinear mixed-effect modeling, unbound mycophenolic acid, linear protein binding, adult kidney transplant recipients

## Abstract

**Objectives:**

The population pharmacokinetic (popPK) characteristics of total mycophenolic acid (tMPA) have been investigated in various ethnic populations. However, investigations of popPK of unbound MPA (uMPA) are few. Thus, a popPK analysis was performed to: (1) characterize the PK of uMPA and tMPA and its 7-O-mycophenolic acid glucuronide (MPAG) metabolite in kidney transplant patients cotreated with cyclosporine (CsA), and (2) identify the clinically significant covariates that explain variability in the dose–exposure relationship.

**Methods:**

A total of 740 uMPA, 741 tMPA, and 734 total MPAG (tMPAG) concentration–time data from 58 Chinese kidney transplant patients receiving MPA in combination with CsA were analyzed using NONMEM^®^ software with the stochastic approximation expectation maximization (SAEM) followed by the important sampling (IMP) method. The influence of covariates was tested using a stepwise procedure.

**Results:**

The PK of uMPA and unbound MPAG (uMPAG) were characterized by a two- and one-compartment model with first-order elimination, respectively. A linear protein binding model was used to link uMPA and tMPA. Apparent clearance (CL/F) and central volume of distribution (V_C_/F) of uMPA (CL_uMPA_/F and V_CuMPA_/F, respectively) and protein binding rate constant (*k*_B_) were estimated to be 851 L/h [relative standard error (RSE), 7.1%], 718 L (18.5%) and 53.4/h (2.3%), respectively. For uMPAG, the population values (RSE) of CL/F (CL_uMPAG_) and V_C_/F (V_CuMPAG_/F) were 5.71 L/h (4.4%) and 29.9 L (7.7%), respectively. Between-subject variability (BSVs) on CL_uMPA_/F, V_CuMPA_/F, CL_uMPAG_/F, and V_CuMPAG_/F were 51.0, 80.0, 31.8 and 48.4%, respectively, whereas residual unexplained variability (RUVs) for uMPA, tMPA, and uMPAG were 47.0, 45.9, and 22.0%, respectively. Significant relationships were found between *k*_B_ and serum albumin (ALB) and between CL_uMPAG_/F and glomerular filtration rate (GFR). Additionally, model-based simulation showed that changes in ALB concentrations substantially affected tMPA but not uMPA exposure.

**Conclusions:**

The established model adequately described the popPK characteristics of the uMPA, tMPA, and MPAG. The estimated CL_uMPA_/F and unbound fraction of MPA (FU_MPA_) in Chinese kidney transplant recipients cotreated with CsA were comparable to those published previously in Caucasians. We recommend monitoring uMPA instead of tMPA to optimize mycophenolate mofetil (MMF) dosing for patients with lower ALB levels.

## Introduction

Mycophenolate mofetil (MMF), a prodrug of mycophenolic acid (MPA), is the predominant antimetabolite immunosuppressant used as a cotherapy with tacrolimus (TAC) or cyclosporine (CsA) to prevent graft rejection after solid organ transplantation (Hart et al., 2018; Hart et al., 2019). MMF is extensively absorbed and rapidly hydrolyzed to the active component MPA after oral administration. The majority of MPA is metabolized to the pharmacologically inactive 7-O-mycophenolic acid glucuronide (MPAG), whereas a lower fraction is metabolized to the active acyl-glucuronide mycophenolic acid (AcMPAG) (Bullingham et al., 1998; Kiang and Ensom, 2016). MPA and MPAG are reported to be 97 and 82% bound to serum albumin (ALB), respectively at clinically relevant concentrations (Bullingham et al., 1998). MPAG also undergoes enterohepatic circulation (EHC) through biliary excretion, followed by intestinal deglucuronidation and reabsorption as MPA in the colon. This process contributes to approximately 40% (range: 10–60%) of the area under the concentration–time curve (AUC) of MPA and causes multiple peaks in the concentration–time profile (Staatz and Tett, 2007). Most absorbed MMF is eliminated through the kidney as MPAG (Staatz and Tett, 2007).

MPA has a narrow therapeutic window and it is recommended to maintain a 12-h dosing interval exposure (AUC_0–12h_) between 30 and 60 mg·h/L during the early posttransplantation period (Shaw et al., 2001; van Gelder et al., 2006; Kuypers et al., 2010; Le Meur et al., 2011). Under-exposure is associated with an increased risk for acute rejection, whereas a higher AUC_0–12h_ may lead to over-immunosuppression. Large between-subject variability (BSV) and time-dependent variation within-subjects are characteristics of MPA pharmacokinetics (PK) (Shaw et al., 2003; Le Meur et al., 2007; van Hest et al., 2007). A 10-fold variation of MPA exposure was observed even in subjects administered the same dose during the first 2 weeks following kidney transplantation. Moreover, the MPA exposure in the early phase posttransplantation was 30–50% lower than that in the stable period when administered the same MMF dose (Shaw et al., 2003).

The narrow therapeutic window and large PK variability make it necessary to individualize MMF therapy based on therapeutic drug monitoring. Currently, the maximum *a posterior* Bayesian method using population PK (popPK) in combination with Bayesian estimation is recommended for facilitating the optimal pharmacotherapy (Tobler and Muhlebach, 2013; Wright and Duffull, 2013; Zhao et al., 2016; Mao et al., 2018). This approach is based on a comprehensive understanding of *prior* information, *i.e.*, the popPK characteristics.

The popPK characteristics of total MPA (tMPA) in kidney transplant recipients have been extensively investigated in various ethnic populations. Regarding unbound MPA (uMPA), the pharmacologically active component, only a few investigations have used the population approach (de Winter et al., 2009; van Hest et al., 2009; Colom et al., 2018; Okour et al., 2018) because of the technical complexity of measurements. Furthermore, the information in Chinese kidney transplant recipients is limited. Therefore, the objectives of this study were to develop a popPK model to: (1) characterize the PK of uMPA, tMPA, and the main metabolite MPAG in Chinese kidney transplant patients cotreated with CsA, and (2) identify the clinically significant covariates that explain the variability in the dose–exposure relationship.

## Methods

### Study Design and Patients

The data were obtained from two clinical studies (Jiao et al., 2007; Geng et al., 2012). The inclusion criteria were as follows: patients 1) receiving first-time kidney transplantation; 2) administered triple immunosuppressive therapy comprising MMF (CellCept^®^, Roche Pharma Ltd., Shanghai, China), CsA, and corticosteroids; 3) and aged over 18 years. The exclusion criteria were: 1) pregnant or lactating women; patients 2) with severe gastrointestinal disorders; 3) cotreated with cholestyramine; 4) and receiving combined organ transplantation.

The first study was an evaluation of the PK of MPA and MPAG during the early posttransplantation period conducted at Huashan Hospital, Fudan University (Jiao et al., 2007). MMF was initiated at 1,500 mg/day from the day of surgery. The second study was an open-label, multicenter, two-phase, sequential, bioequivalence study conducted in stable kidney transplantation patients (Geng et al., 2012). MMF dose was 1,000 or 1,500 mg/day in most patients. All protocols were approved by the independent Clinical Research Ethics Committee of Huashan Hospital, Fudan University, and all participants provided written informed consent before enrolment.

After the morning dose, whole blood samples were collected at 0, 0.5, 1, 1.5, 2, 3, 4, 6, 8, 10, and 12 h in study 1 and 0, 0.5, 1, 1.5, 2, 2.5, 3, 4, 6, 9, 10, and 12 h in study 2. Low-fat meals were provided after the scheduled 4 and 10 h samplings in study 1 and the 3 and 9 h samplings in study 2. The relevant data were collected to explore the relationships between demographic characteristics, biochemical measurements, and PK parameters.

All samples were analyzed at Huashan Hospital using a validated high-performance liquid chromatography method (Jiao et al., 2005; Jiao et al., 2007). The calibration ranges were 0.002–1.0, 0.1–40, and 10–200 mg/L for uMPA, tMPA, and total MPAG (tMPAG), respectively. The relative bias at lower limit of quantification (LLOQ) were within ±17% for uMPA, within ±8.3% for tMPA, and tMPAG. The relative bias of other quality control concentrations for uMPA, tMPA, and tMPAG was within ±6.1%. The intra- and interday precision, as coefficient of variation values, were <14% for uMPA, < 9.2% for tMPA, and <9.8% for tMPAG.

### PopPK Analyses

#### Software and Model Selection Criteria

Nonlinear mixed-effect modeling was performed using NONMEM^®^ software (version 7.4; ICON Development Solutions, Ellicott City, MD, USA) compiled with gfortran 4.6.0. Perl-speaks-NONMEM (PsN, version 4.7.0; http://uupharmacometrics.github.io/PsN) and Pirana (version 2.9.7; http://www.certara.com/pirana) were used to link NONMEM, model development, and model evaluation. The stochastic approximation expectation maximization (SAEM), followed by important sampling (IMP) method (Bauer, 2017) were used throughout the model development. Graphical diagnostics were performed using R software (version 3.4.4, http://www.r-project.org).

MMF doses and uMPA, tMPA, and tMPAG concentrations were transformed into molar equivalents by dividing them with the molecular weight (MMF, MPA, and MPAG: 433.498, 320.339, and 496.462 g/mol, respectively; http://chem.nlm.nih.gov/chemidplus/) and then reconverted to milligram per liter in the figures and results. Model selection was based on goodness-of-fit (GOF) plots (Ette and Ludden, 1995) in addition to the three commonly used criteria of statistical significance, plausibility, and stability. The difference in objective function values (OFV) between two nested models was used for statistical comparison. Akaike information criteria (AIC) (Vaida and Blanchard, 2005) and Bayesian information criteria (BIC) (Delattre et al., 2012) were used to discriminate nonnested models.Additionally, relative standard errors (RSEs) of parameter estimates, shrinkages, and changes of BSV and residual unexplained variability (RUV) estimates were considered. During the model developing process, the condition numbers were calculated and no more than 1,000 were kept to avoid overparameterization (Owen and Fiedler-Kelly, 2014).

#### Model Development

PopPK modeling of MPA and MPAG was conducted using a sequential approach and eventually led to simultaneous modeling of both the parent compound and metabolite. One- or two-compartments models with first-order elimination were tested for uMPA and unbound MPAG (uMPAG). We further investigated whether MPA absorption was best described by a first- or zero-order process, with or without a lagged absorption time (Tlag). The concentrations of uMPAG were not determined in our study but were estimated from tMPAG by multiplying the unbound fraction of MPAG (FU_MPAG_), which was fixed at 18% according to the U.S. Food and Drug Administration (FDA) package insert for CellCept^®^ (FDA, 2019).

The tMPA data was first modeled by adding a linear protein binding compartment as equation 1:

(1) $$C_{\mathrm{tMPA}}=C_{\mathrm{uMPA}}+k_{B}\times C_{\mathrm{uMPA}}$$

where C_tMPA_ and C_uMPA_ represent total and unbound MPA concentrations, respectively, and *k*_B_ is the protein binding rate constant. In this case, the unbound fraction of MPA (FU_MPA_) could be expressed as equation 2:

(2) $$FU_{\mathrm{MPA}}=\frac{C_{\mathrm{uMPA}}}{C_{\mathrm{tMPA}}}=\frac{1}{1+k_{B}}$$

The nonlinear saturable protein binding model published previously (Picard-Hagen et al., 2001; Colom et al., 2018) was also evaluated using equation 3:

(3) $$C_{\mathrm{bMPA}}=\frac{B_{\max}\times C_{\mathrm{uMPA}}}{k_{D}+C_{\mathrm{uMPA}}}$$

where C_bMPA_ represents the bound MPA concentration, B_max_ is the maximal number of protein binding sites, and *k*_D_ is the dissociation constant representing the uMPA concentration corresponding to half-saturation of protein binding. To describe the physiological EHC process, the previously published intermittent EHC model (Jiao et al., 2008; Ling et al., 2015) was used with some modifications, in which a gallbladder compartment was introduced to connect MPAG and gut compartments. The percentage of MPAG recycled into the systemic circulation (%EHC) was described using equation 4:

(4) $$\%\mathrm{EHC}=\frac{k_{\mathrm{GG}}}{k_{\mathrm{GG}}+k_{e0}}\times 100$$

where, *k*_GG_ is the transfer rate constant from the MPAG central compartment to the gallbladder and *k*_e0_ is the elimination rate constant of MPAG.

Several assumptions were made to ensure the model was structurally identifiable (Jiao et al., 2008): (1) MMF is quickly absorbed and completely hydrolyzed to MPA, (2) the conversion ratio from MPA to MPAG is fixed at 87% (FDA, 2019), (3) MPAG secreted from the gallbladder to the intestines is completely deconjugated to MPA and reabsorbed, (4) the rate constants associated with each compartment are all first-order and unaffected by the recycling, and (5) gallbladder emptying is triggered by meals. Additionally, the gallbladder emptying rate constant (*k*_GB_) was fixed at 3.708/h based on previous study (Guiastrennec et al., 2016). The duration (D_GB_) of gallbladder release was fixed at 0.5 h to ensure that over 90% gallbladder contents would be released after each trigger.

An exponential model was used to describe BSVs for each PK parameter while exponential, additive, and combined models were compared to describe RUVs. Furthermore, the covariance of BSVs was estimated using OMEGA BLOCK statement in NONMEM. Because uMPA, tMPA, and tMPAG concentrations were derived from the same sample for each subject, their RUVs were likely correlated. An L2 data item was introduced and the covariance of RUVs was also evaluated using SIGMA BLOCK statement (Bauer, 2017).

After the base model was established, the following physiologically meaningful covariates were investigated: sex, age, body weight (BW), postoperative time (POT), hemoglobin, ALB, alanine aminotransferase, aspartate aminotransferase, serum creatinine (SCr), glomerular filtration rate (GFR), CsA daily dose, and coadministration of antacids. GFR was estimated from SCr using the Chronic Kidney Disease Epidemiology Collaboration formula (Levey et al., 2009).

First, relationships between individual PK parameters and covariates were examined by graphical inspection to identify the potential covariates. Then, the identified covariates were tested using a stepwise procedure. During the forward inclusion and backward elimination steps, significance levels were set at a decrease in OFV > 3.84 (χ^2^, *df* = 1, *p* < 0.05) and an increase in OFV > 10.83 (χ^2^, *df* = 1, *p* < 0.001), respectively. The continuous covariates were assessed using a linear and non-linear model, and categorical covariates were modeled proportionally. To demonstrate clinical significance, covariates were only retained if the effect on the corresponding parameter was >15% for a categorical covariate, or >15% at the highest or lowest observed covariate value for a continuous covariate (Mo et al., 2018). In addition, the included covariates were expected to have interpretations of physiological or pharmacological mechanisms.

#### Model Evaluation

The established model was evaluated by graphical diagnosis. GOF plots included scatterplots of population predictions (PRED) and individual predictions (IPRED) *versus* observed concentrations (OBS), as well as conditional weighted residuals (CWRES) *versus* PRED and time after previous dose (TAD). Observations over ±4 CWRES based on final model were excluded from the original dataset, and the sensitivity analysis was performed to verify the model. Additionally, 500 bootstraps (Ette et al., 2003) were applied to assess the reliability and stability of the final model. The medians and 2.5–97.5% intervals from the bootstrap replicates were compared with estimates of the final model.

The final model was further examined using a prediction-corrected visual predictive check (pc-VPC) (Bergstrand et al., 2011) and posterior predictive check (PPC) (Yano et al., 2001). Furthermore, 2,000 datasets were simulated using the final model from the original dataset. For pc-VPC, the observed and simulated concentrations were dose-normalized to 750 mg MMF every 12 h. The median, 5th and 95th percentiles of simulated concentrations and corresponding 95% confidence intervals (CIs) were calculated and graphically compared with the observations. PPC was further performed to assess if the model appropriately predicted the AUC_0–12h_ of uMPA, tMPA, and tMPAG. Simulated and observed AUC_0–12h_ were calculated using the linear trapezoidal rule. Distributions of the simulated and observed AUC_0–12h_ were then graphically compared.

### Simulation Analyses of Effects of Significant Covariates

The established final model was used to investigate the effect of the identified covariates on the PK of MPA and MPAG. Specifically, 2,000 stochastic simulations were performed for virtual subjects administered 750 mg MMF every 12 h with different covariate levels. The AUC_0–12h_ values of uMPA, tMPA, and tMPAG were estimated using the linear trapezoidal rule, and changes in AUC_0–12h_ and FU_MPA_ were assessed.

## Results

### Patient Characteristics and Data Descriptions

A total of 27 full concentration–time profiles containing uMPA, tMPA, and tMPAG data were obtained from 20 patients in study 1, including 23 profiles collected within 3 months posttransplantation. Sixteen patients had one profile, one had two profiles, and the other three each had three profiles. In study 2, we obtained 38 full concentration–time profiles from 38 patients, including 37 collected beyond 3 months posttransplantation. The patient characteristics are shown in **Table 1**. Of these subjects, male patients accounted for approximately 78%. The concomitant antacids in study 1 were proton pump inhibitors, whereas sodium hydrogen carbonate and compound aluminum hydroxide were coadministered in study 2. Significant differences in BW, POT, hemoglobin, and ALB as well as doses of MMF, CsA, and corticosteroids were observed between the two studies.

**Table 1:** Patient characteristics and clinical covariates.

Characteristics	Study 1		Study 2		*P* value^a^
	median (range)	mean ± SD	median (range)	mean ± SD	
Patients, n	20	/	38	/	/
Sex					
Male, n (%)	11 (55)	/	34 (89)	/	< 0.01
Female, n (%)	9 (45)	/	4 (11)	/	< 0.01
Age, years	36 (19–61)	37 ± 12	38 (18–62)	38 ± 12	> 0.05
Body weight, kg	55 (40–71)	54.3 ± 9.8	65 (42–82.5)	65.2 ± 10.2	< 0.001
Postoperative time, days	10 (3–148)	31 ± 41	298 (70–3084)	620 ± 780	< 0.001
Mycophenolate mofetil daily dose, mg/day	1,500 (750–2,000)	1,444 ± 313	1,000 (1,000–2,000)	1230 ± 269	< 0.01
Hemoglobin, g/L	86 (72–134)	93.6 ± 18.6	139 (103–181)	142.6 ± 22.4	< 0.001
Albumin, g/L	31 (20–43)	32 ± 6.6	44.9 (32.3–50)	44.2 ± 3.9	< 0.001
Alanine aminotransferase, U/L	24 (10–390)	49.48 ± 78.51	18 (7–64)	21.88 ± 12.65	> 0.05
Aspartate aminotransferase, U/L	20 (7–139)	33.78 ± 29.32	24 (8.6–86)	28.94 ± 19.88	> 0.05
Serum creatinine, μmol/L	96 (50–443)	114.41 ± 73.97	104.5 (76–152.9)	108.82 ± 17.27	> 0.05
Glomerular filtration rate^b^, mL/min	76.12 (11.17–123.8)	75.58 ± 25.09	74.42 (45.14–102.3)	74.79 ± 14.16	> 0.05
**Concomitant medication**					
Cyclosporine daily dose, mg/day	300 (0–400)	282 ± 102	220 (100–400)	231 ± 65	< 0.01
Corticosteroid daily dose, mg/day	20 (5–675)	49.1 ± 126.1	10 (3–20)	10.8 ± 4.1	< 0.001
Antacids^c^, n (%)	6 (22)	/	5 (13)	/	> 0.05
Aspirin, n (%)	0 (0)	/	6 (16)	/	< 0.05
Nifedipine, n (%)	4 (15)	/	5 (13)	/	> 0.05
Diltiazem, n (%)	0 (0)	/	7 (18)	/	< 0.05

/, not applicable; SD, standard deviation.

a Differences between groups are determined using the Mann–Whitney U test for continuous variables and Fisher’s exact test for categorical data with IBM SPSS Statistics for Windows (Version 20, IBM Corp., Armonk, NY).

b Glomerular filtration rate (GFR) is calculated from serum creatinine using the Chronic Kidney Disease Epidemiology Collaboration formula (Levey et al., 2009): GFR = 141 × min(SCr/κ, 1)^α^ × max(SCr/κ, 1)^−1.209^ × 0.993^Age^ × 1.018 [if female] × 1.159 [if black], where SCr is serum creatinine, κ is 62 (μmol/L) for females and 80 (μmol/L) for males, α is −0.329 for females and −0.411 for males, min indicates the minimum of SCr/κ or 1, and max indicates the maximum of SCr/κ or 1.

c Antacids include proton pump inhibitors, sodium hydrogen carbonate, and compound aluminum hydroxide.

Of the 2,229 samples, < 1% (3 uMPA, 1 tMPA, and 10 tMPAG) were below the LLOQ and were discarded. In total, 740 uMPA, 741 tMPA, and 734 tMPAG concentration measurements were used for the popPK analysis. Multiple uMPA and tMPA peaks attributed to EHC were observed at 4–6 and 8–12 h postdosing in some subjects, whereas no obvious multiple peaks were observed for tMPAG. After being normalized to MMF 1,500 mg/day, the median AUC_0–12h_ of uMPA in study 2 was significantly higher than that in study 1 (38.60 *vs.* 27.21 mg·h/L). No significant differences in the AUC_0–12h_ of tMPA and tMPAG were found between the two studies.

### PopPK Model

#### Model Development

**Figure 1:**
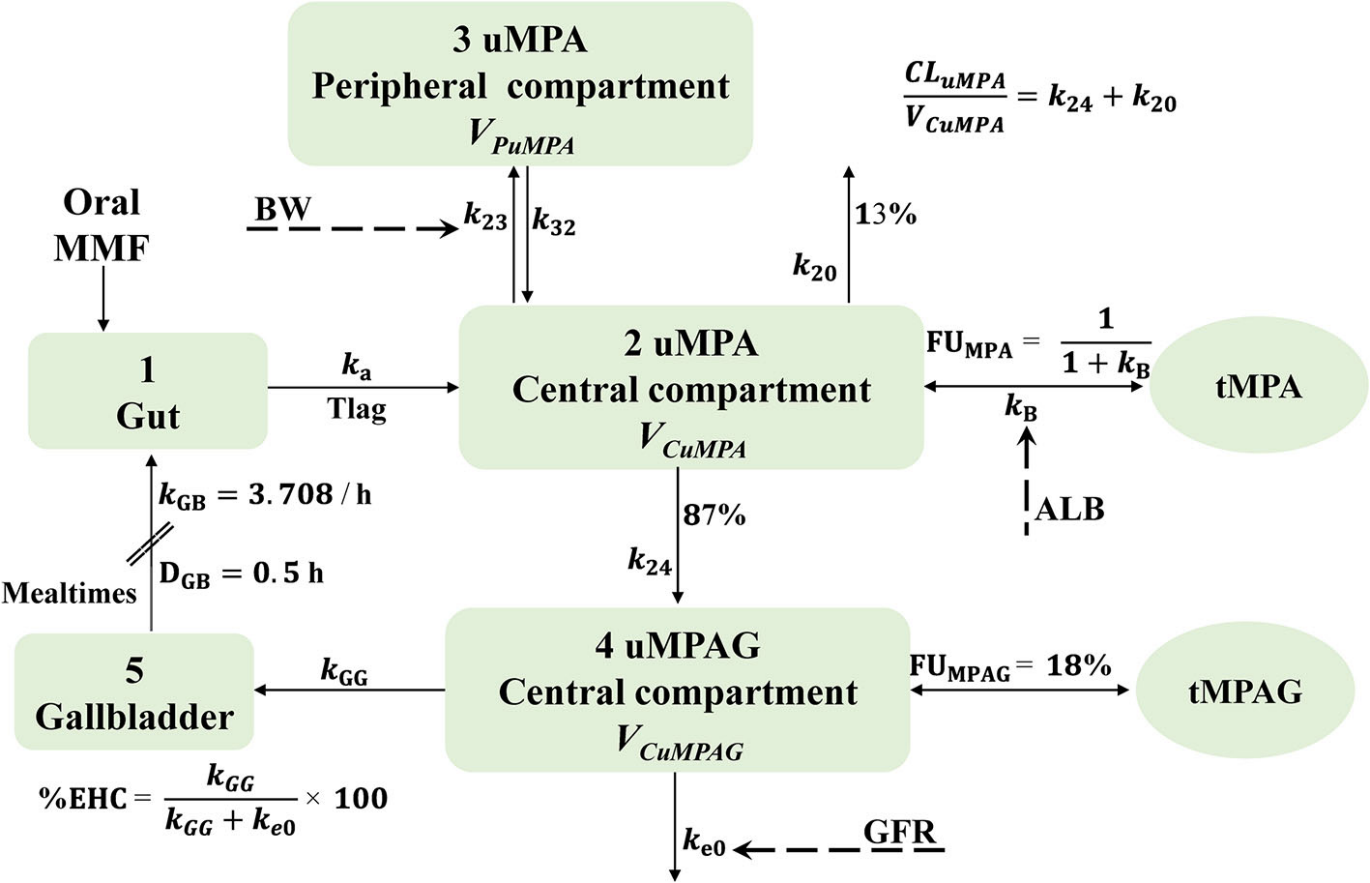
Schematic representation of the final structural model characterizing the linear protein binding and intermittent EHC processes. In this model, mealtimes are used as an index of gallbladder emptying. This process is assumed to occur at specific time points (mealtimes) with a first-order rate constant and a certain duration. The fraction of MPA metabolized to MPAG is fixed at 87%. MMF, mycophenolate mofetil; MPA, mycophenolic acid; MPAG, 7-O-mycophenolic acid glucuronide; tMPA, total MPA; tMPAG, total MPAG; uMPA, unbound MPA; uMPAG, unbound MPAG; ALB, serum albumin; BW, body weight; CL_uMPA_/F, apparent clearance of uMPA; D_GB_, duration of gallbladder emptying; EHC, enterohepatic circulation; %EHC, percentage of MPAG recycled into the systemic circulation; FU_MPA_, unbound fraction of MPA; FU_MPAG_, unbound fraction of MPAG; GFR, glomerular filtration rate; *k*_20_, elimination rate constant of uMPA; *k*_23_, transfer rate constant from uMPA central compartment to peripheral compartment; *k*_24_, rate constant of uMPA transformed to uMPAG; *k*_32_, transfer rate constant from uMPA peripheral compartment to central compartment; *k*_a_, absorption rate constant; *k*_B_, protein binding rate constant; *k*_e0_, elimination rate constant of uMPAG; *k*_GB_, gallbladder emptying rate constant; *k*_GG_, transfer rate constant from uMPAG central compartment to gallbladder; Tlag, lagged absorption time; V_CuMPA_/F and V_CuMPAG_/F, apparent central volume of distribution of uMPA and uMPAG, respectively; V_PuMPA_/F, apparent peripheral volume of distribution of uMPA.

As shown in **Figure 1**, a five-compartment model with first-order absorption and elimination adequately described the uMPA, tMPA, and uMPAG data. The two-compartment (2CMT) structural model was superior to the one-compartment (1CMT) (AIC, −2904.957 *vs.* −2585.189; BIC, −2785.184 *vs.* −2506.876) for uMPA. Moreover, the 1CMT structural model showed a better fit for uMPAG than the 2CMT did (AIC, −81.269 *vs.* −59.793; BIC, 188.813 *vs.* 379.753). Incorporation of Tlag further led to a significant reduction of 383.714 units in the OFV. Simultaneous estimation of both B_max_ and *k*_D_ was not feasible; therefore, B_max_ was fixed at the reported value of 35,100 μmol (de Winter et al., 2009). The nonlinear saturable binding from the central compartment did not improve the fit (AIC, 1531.731 *vs.* 1530.953; BIC, 2010.784 *vs.* 2010.006) more than the linear protein binding model did. Furthermore, incorporation of the EHC process decreased the AIC and BIC by 129.985 and 21.628 units, respectively.

The PK parameters estimated were absorption rate constant (*k*_a_), Tlag, apparent clearance (CL/F) of uMPA and uMPAG (CL_uMPA_/F and CL_uMPAG_/F, respectively), apparent intercompartmental clearance of uMPA (Q_uMPA_/F), apparent central volume of distribution (V_C_/F) of uMPA and uMPAG (V_CuMPA_/F and V_CuMPAG_/F, respectively), *k*_B_, and %EHC. Apparent peripheral volume of distribution of uMPA (V_PuMPA_/F) could not be estimated appropriately and was fixed at the reported literature value of 34,300 L (de Winter et al., 2009).

Considering that the expectation maximization (EM) algorithm is much more robust and adept at handling the large full OMEGA block (Bauer, 2017), we initially attempted to assign BSVs to all PK parameters. However, our data did not support the estimation of BSV on *k*_B_. To maximally enhance the EM efficiency, the BSV was assigned to *k*_B_, and its variance was fixed at 0.01 (Bauer, 2017). Various RUV models were tested to describe the residual errors. Incorporation of the additive residual error resulted in boundary issues and therefore, an exponential RUV model was used.

Based on the visual inspections and clinical plausibility, the effects of BW and sex on CL_uMPA_/F, Q_uMPA_/F, V_CuMPA_/F, CL_uMPAG_/F, and V_CuMPAG_/F; GFR on *k*_B_, CL_uMPA_/F, Q_uMPA_/F, and CL_uMPAG_/F; ALB on *k*_B_, V_CuMPA_/F, and V_CuMPAG_/F; coadministration of antacids on CL_uMPA_/F and *k*_a_; and tMPAG concentrations on *k*_B_ were further tested using the stepwise method. Of these, the effects of ALB on *k*_B_, GFR on CL_uMPAG_/F, and BW and sex on Q_uMPA_/F were included in the forward procedure, whereas the effect of sex on Q_uMPA_/F showed no significance in the backward step and, thus, was not retained in the final model. The forward inclusion and backward elimination steps are summarized in **Supplementary Table 1**. Because the estimated value of the exponent for the effect of ALB on *k*_B_ was quite close to 1, it was fixed at 1 to simplify the model and maintain the model stability.

Although introduction of full variance–covariance matrices for BSVs and RUVs substantially decreased the OFV by 262.265 units, the high condition number (7.5 × 10^10^) indicated that the model might be ill-conditioned because of overparameterization. Finally, the covariance between BSVs for V_CuMPAG_/F and CL_uMPAG_/F and between RUVs for uMPA and tMPA was included. This further decreased the OFV by 163.434 units with an acceptable condition number (< 150). The parameter estimates of the final model are provided in **Table 2**. No significant covariate was detected to influence CL_uMPA_/F, whereas significant relationships were found between *k*_B_ and ALB and between CL_uMPAG_/F and GFR. RSEs of the parameter estimates were <30 and 45% for fixed and random effects except for %EHC, respectively. Shrinkage values of BSVs and RUVs were <30% except for %EHC.

**Table 2:** Pharmacokinetic parameter estimates for the final model and Bootstrap results.

Parameters	Estimates	%RSE^a^	Shrinkage(%)	Bootstrap			
				Median			2.5th–97.5th percentile^b^
*Pharmacokinetic parameters for uMPA and uMPAG*							
CL_uMPA_/F, L/h	851	7.1	/	855			723–1,012
Q_uMPA_/F^c^, L/h	857	11.0	/	843			710–1,018
Exponent for the effect of BW on Q_uMPA_/F	2.11	24.2	/	2.06			1.02–3.10
V_CuMPA_/F, L	718	18.5	/	710			492–937
* k*_a_,/h	1.35	11.1	/	1.34			1.11–1.61
Tlag, h	0.447	16.8	/	0.449			0.297–0.602
* k*_B_^d^,/h	53.4	2.3	/	53.5			45.3–61.6
CL_uMPAG_/F^e^, L/h	5.71	4.4	/	5.72			5.18–6.51
Exponent for the effect of GFR on CL_uMPAG_/F	0.865	11.6	/	0.849			0.320–1.580
V_CuMPAG_/F, L	29.9	7.7	/	30.0			26.0–35.0
%EHC	5.53	26.2	/	5.87			3.49–8.83
*Pharmacokinetic parameters for tMPA and tMPAG ^f^*							
CL_tMPA_/F, L/h	15.66	/	/	/			/
Q_tMPA_/F, L/h	15.77	/	/	/			/
V_CtMPA_/F, L	13.21	/	/	/			/
V_PtMPA_/F, L	631.12	/	/	/			/
CL_tMPAG_/F, L/h	1.03	/	/	/			/
V_CtMPAG_/F, L	5.38	/	/	/			/
*Between-subject variability, %CV*							
CL_uMPA_/F	51.0	11.0	3.6		49.8	39.5–59.5	
Q_uMPA_/F	45.5	16.2	17.8		42.3	27.0–56.3	
V_CuMPA_/F	80.0	25.2	26.1		81.5	53.3–109.5	
* k*_a_	46.5	20.4	27.6		44.1	32.1–59.3	
Tlag	107.7	15.8	8.4		109.5	83.6–151.7	
* k*_B_	10.0 FIXED	/	/		/	/	
CL_uMPAG_/F	31.8	13.3	2.0		32.3	24.7–43.2	
Correlation between CL_uMPAG_/F and V_CuMPAG_/F	57.4	28.7	/		57.5	30.0–79.7	
V_CuMPAG_/F	48.4	25.0	15.5		46.7	27.2–65.2	
%EHC	61.6	55.9	57.7		55.1	15.5–98.8	
*Residual unexplained variability, %CV*							
uMPA	47.0	3.5	5.1		46.7	41.3–52.2	
Correlation between uMPA and tMPA	51.2	7.2	/		51.1	38.2–62.2	
tMPA	45.9	3.7	5.2		45.4	41.0–50.0	
uMPAG	22.0	3.1	4.7		21.2	18.1–24.0	

MPA, mycophenolic acid; MPAG, 7-O-mycophenolic acid glucuronide; tMPA, total MPA; tMPAG, total MPAG; uMPA, unbound MPA; uMPAG, unbound MPAG; /, not applicable; %CV, percentage coefficient of variation; %EHC, percentage of MPAG recycled into the systemic circulation; %RSE, percentage relative standard error; ALB, serum albumin; BW, body weight; CL_tMPA_/F, CL_tMPAG_/F, CL_uMPA_/F and CL_uMPAG_/F, apparent clearance of tMPA, tMPAG, uMPA and uMPAG, respectively; GFR, glomerular filtration rate; k_a_, absorption rate constant; k_B_, protein binding rate constant; Q_tMPA_/F and Q_uMPA_/F, apparent intercompartmental clearance of tMPA and uMPA, respectively; Tlag, lagged absorption time; V_CtMPA_/F, V_CtMPAG_/F, V_CuMPA_/F and V_CuMPAG_/F, apparent central volume of distribution of tMPA, tMPAG, uMPA and uMPAG, respectively; V_PtMPA_/F, apparent peripheral volume of distribution of tMPA.

a %RSE is estimated as the standard error of the estimate divided by the population estimate multiplied by 100.

b Based on 500 successful bootstrap runs.

c The effect of BW on QuMPA/F is expressed as: $Q_{uMPA}/F=857\times {\left[\frac{BW\;(kg)}{70}\right]}^{2.11}\;(L/h)$.

d The effect of ALB on kB is expressed as: $k_{B}=53.4\times \left[\frac{ALB\;(g/L)}{40}\right]\;(/h)$.

e The effect of GFR on CLuMPAG/F is expressed as: $CL_{uMPAG}/F=5.71\times {\left[\frac{GFR\;(mL/\min)}{80}\right]}^{0.865}\;(L/h)$.

f The disposition parameter estimates for tMPA and tMPAG concentrations are generated by multiplying the unbound concentration based parameters in the original model by the typical unbound fraction at serum albumin concentration of 40 g/L.

#### Model Evaluation

**Figure 2:**
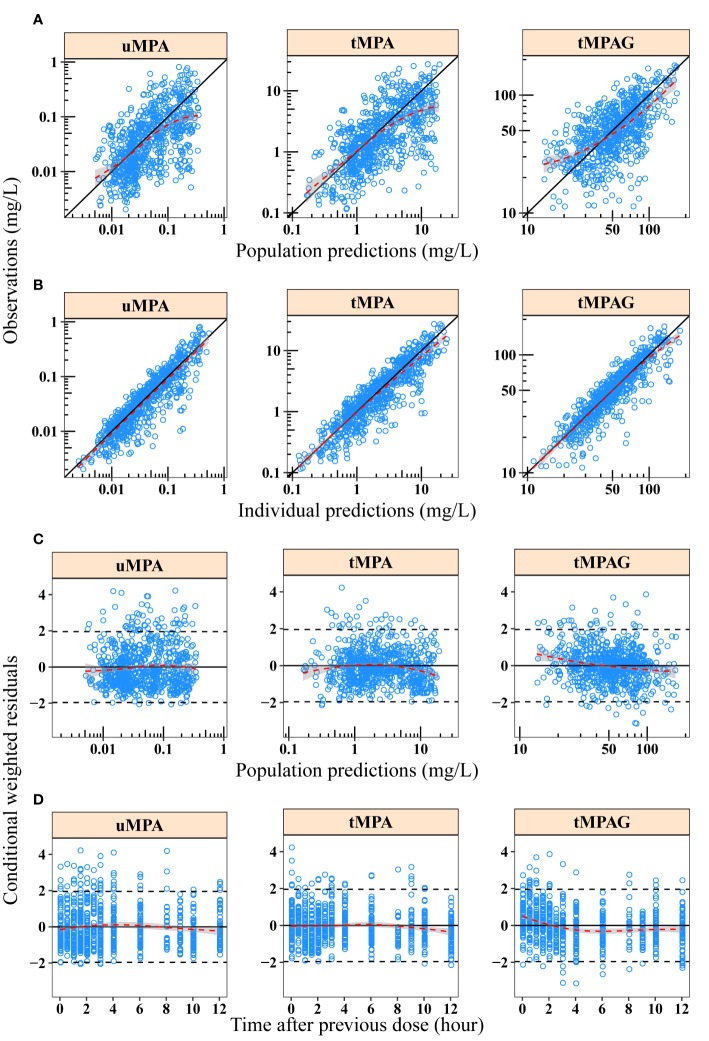
Goodness-of-fit plots of final model for uMPA, tMPA and tMPAG. **(A)** Population predictions *versus* observations; **(B)** individual predictions *versus* observations; **(C)** population predictions *versus* conditional weighted residuals; **(D)** time after previous dose *versus* conditional weighted residuals. Red dashed lines and gray-shaded areas represent the locally weighted regression line and 95% confidence interval, respectively. In plots A and B, black solid lines represent the line of unity. In plots C and D, black solid and dashed lines represent the y = 0 and y = ± 1.96 reference lines, respectively. tMPA, total mycophenolic acid (MPA); tMPAG, total 7-O-mycophenolic acid glucuronide; uMPA, unbound MPA.

The basic GOF plots of the final model are shown in **Figure 2** where PRED and IPRED did not show obvious bias when plotted against OBS. Over 99.8% (2,211/2,215) of the observations were within ±4 CWRES. Alterations of all parameter estimates were < ± 15% when observations with CWRES of > ± 4 were excluded (**Supplementary Table 2**). The GOFs and sensitivity analysis results showed that the model adequately described the data despite the fact that a slightly positive bias could be found in the residual plots.

**Figure 3:**
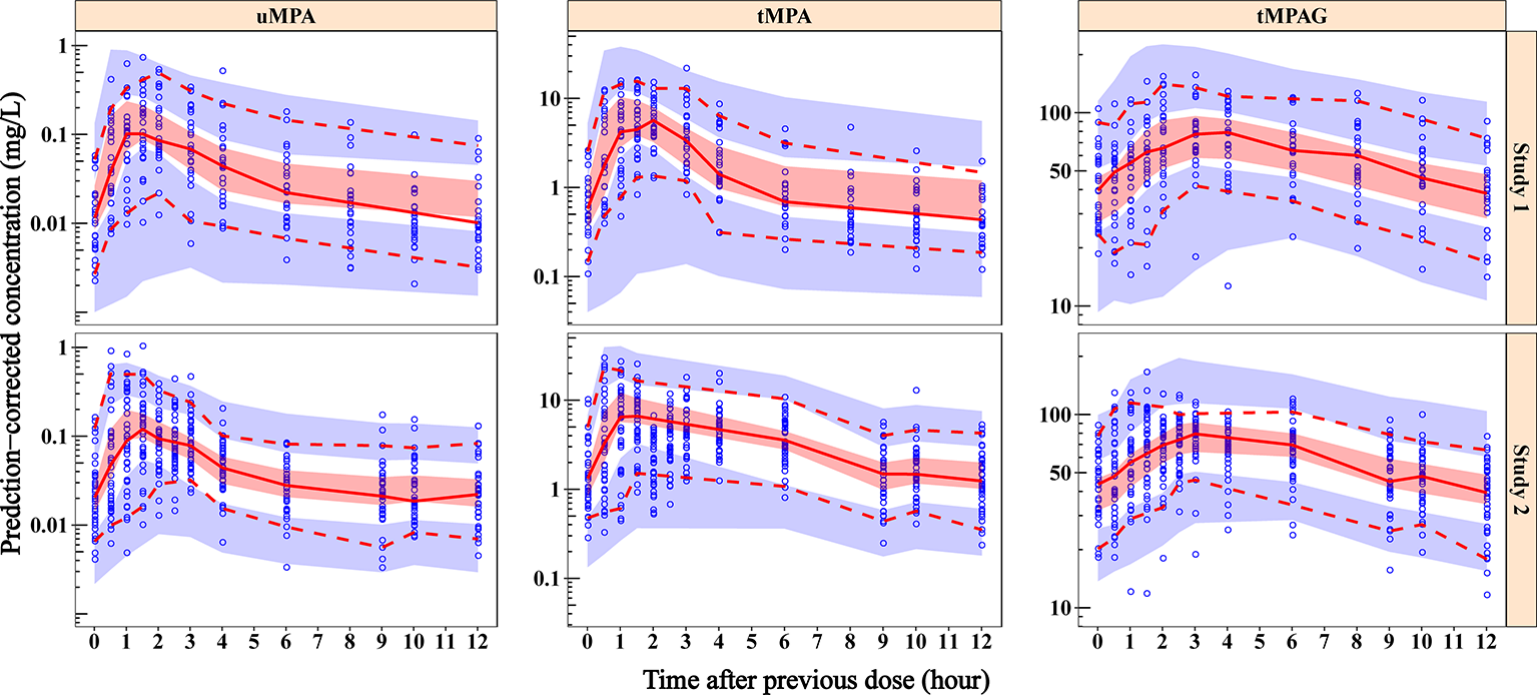
Prediction-corrected visual predictive check plots of final model for uMPA, tMPA and tMPAG. Blue dots represent the observed concentrations. Red solid lines represent the median of observations, and the semitransparent red fields represent the simulation-based 95% CIs for the median. The observed 5th and 95th percentiles are presented with red dashed lines, and the simulation-based 95% CIs for corresponding percentiles are shown as semitransparent blue fields. In general, the median, and 5th and 95th percentile lines of observations fall inside the area of the corresponding 95% CIs. Additionally, the majority of observed concentrations fall within the 90% prediction interval, which demonstrates that the predicted variability does not exceed the observed variability. CIs, confidence intervals; tMPA, total mycophenolic acid (MPA); tMPAG, total 7-O-mycophenolic acid glucuronide; uMPA, unbound MPA.

**Figure 4:**
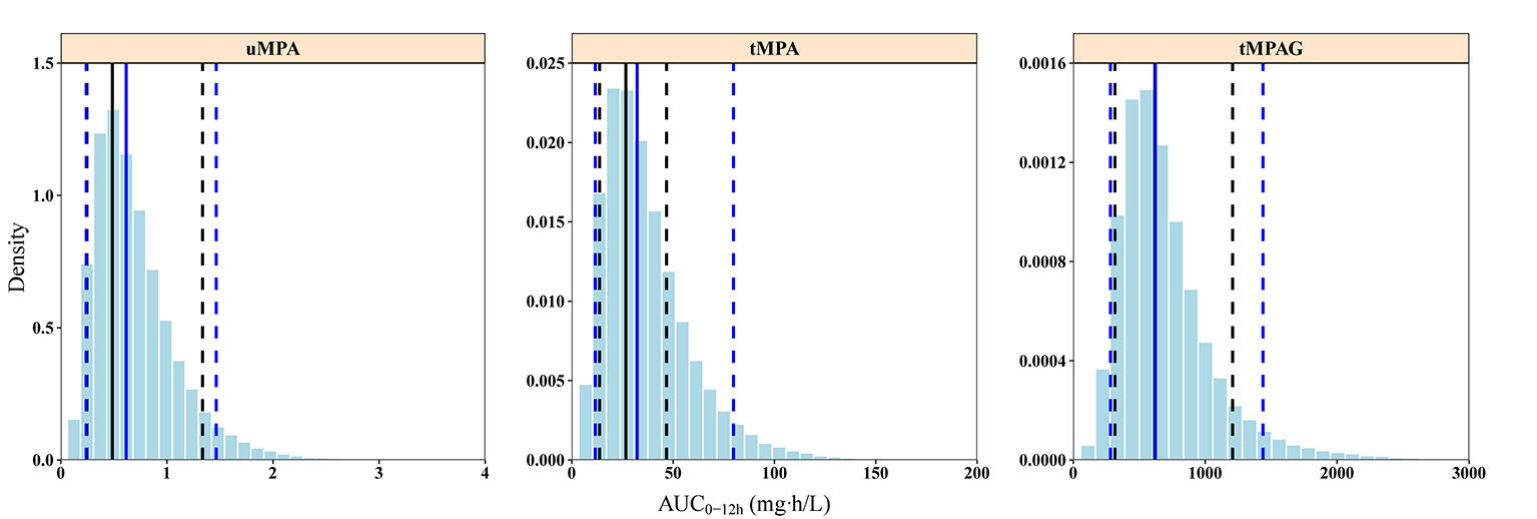
Posterior predictive check graphics of final model for uMPA, tMPA, and tMPAG. The histograms represent the distribution of simulations. Black and blue solid lines represent the medians of observations and simulations, respectively. The observed 5th and 95th percentiles are presented by black dashed lines, and the simulated 5th and 95th percentiles are presented by blue dashed lines. The simulated AUC_0–12h_ values present good consistency with observations. In particular, the 5th percentiles of simulations and observations for uMPA, as well as the medians of simulations and observations for tMPAG, are completely overlapped in the graphics. AUC_0–12h_, area under the concentration–time curve within 12-h dose-interval; tMPA, total mycophenolic acid (MPA); tMPAG, total 7-O-mycophenolic acid glucuronide; uMPA, unbound MPA.

Out of 500 replicates in the bootstrap analysis, all runs converged successfully. The estimated parameters based on original dataset were in good agreement with the median bootstrap replicates and were within the 2.5–97.5% intervals obtained from the bootstrap analysis (**Table 2**), indicating the reliability and stability of the final model. **Figure 3** shows the results of the pc-VPC of the final model. Most observed concentrations fell within the 90% prediction interval, and no obvious discrepancy between observations and simulations was found. The PPC suggested that the simulated AUC_0–12h_ values also showed good consistency with the observations (**Figure 4**). The pc-VPC and PPC results showed that the final model was reasonably good at predicting the observations.

### Simulations Illustrating Effect of Covariates

**Figure 5:**
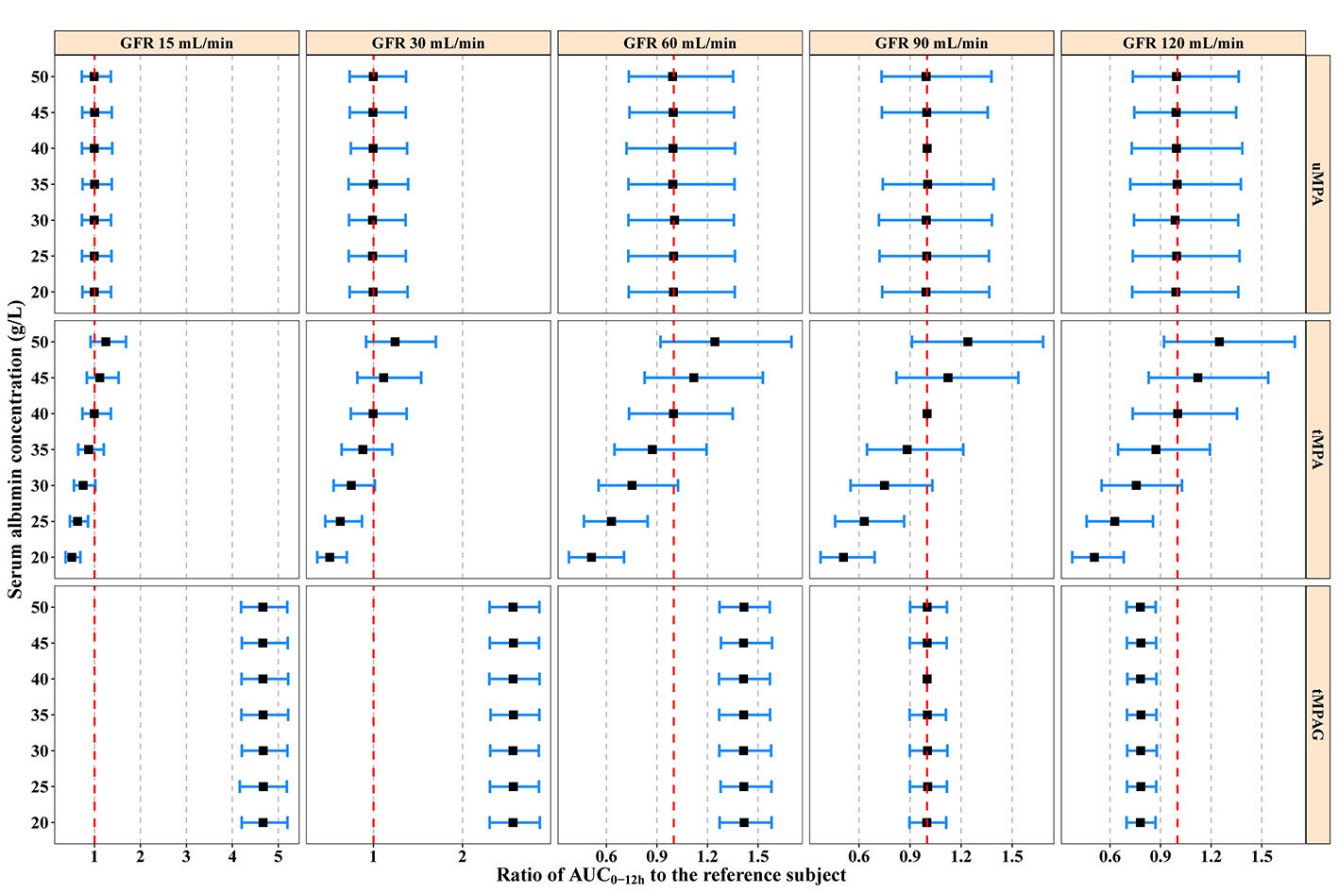
Model-predicted covariate effects on AUC_0–12h_ of uMPA, tMPA, and tMPAG. Black squares represent median values and error bars represent 95% confidence intervals of the normalized exposure ratios relative to the typical reference subject (ALB 40 g/L, GFR 90 mL/min) across 2,000 simulation replicates. The vertical red dashed lines show an exposure ratio of 1 relative to the reference subject. ALB, serum albumin; AUC_0–12h_, area under the concentration–time curve within 12-h dose-interval; GFR, glomerular filtration rate; tMPA, total mycophenolic acid (MPA); tMPAG, total 7-O-mycophenolic acid glucuronide; uMPA, unbound MPA.

Typical subjects administered 750 mg MMF every 12 h were simulated with different ALB and GFR levels. ALB values were set from 20 to 50 g/L with a step of 5 g/L. At each ALB level, the GFR was set at 15, 30, 60, 90, and 120 mL/min according to the Kidney Disease Improving Global Outcomes (KIDGO) chronic kidney disease classification (Kidney Disease: Improving Global Outcomes CKD Work Group, 2013). Generally, ALB and GFR showed large effects on tMPA and tMPAG, respectively, but little effect on uMPA (**Figure 5**).

A substantial decrease in tMPA AUC_0–12h_ and increase in FU_MPA_ were observed with decreasing ALB concentrations. For subjects with a GFR of 90 mL/min administered 750 mg MMF every 12 h, the median tMPA AUC_0–12h_ decreased from 42.92 to 21.59 mg·h/L when ALB concentrations decreased from 40 to 20 g/L, whereas the exposure of uMPA and tMPAG remained almost unchanged (< 5%). A decrease in ALB concentrations from 40 to 20 g/L increased FU_MPA_ from 1.86% (95% CI, 1.35–2.58%) to 3.62% (95% CI, 2.62–5.02%) (**Supplementary Figure 1**).

Additionally, a substantial increase in tMPAG AUC_0–12h_ was observed with decreasing GFR. For subjects with an ALB concentration of 40 g/L administered 750 mg MMF every 12 h, a reduction in GFR from 90 to 15 mL/min led to a 3.67-fold (95% CI, 3.13–4.30) increase in tMPAG AUC_0–12h_, while FU_MPA_ and the exposure of both uMPA and tMPA were unchanged (**Figure 5** and **Supplementary Figure 1**).

The simulations showed that neither ALB nor GFR significantly affected uMPA exposure. For patients with lower ALB levels, dose adjustment based on monitoring tMPA would lead to higher risk of leucopoenia and infections, because of overexposure to uMPA.

## Discussion

The present study extensively investigated the popPK characteristics of uMPA, tMPA, and MPAG in Chinese adult kidney transplant recipients cotreated with CsA during both the early and stable periods posttransplantation. A two-compartment model with first-order absorption and elimination adequately described the uMPA data. Furthermore, the uMPA and tMPA were connected using a linear protein binding model.

It is still controversial whether ethnic differences exist in MPA PK. Some studies have reported higher dose-normalized AUCs of MPA in Asian subjects than in Caucasians (Lu et al., 2005; Zicheng et al., 2006; Yau et al., 2007; Zhou et al., 2007; Miura et al., 2009). Li et al. (2014) reported that Asians had lower MPA CL/F and required lower MMF doses than Caucasians did. This could be partly explained by the significantly lower BW of Asian subjects.

After correcting for BW, the average tMPA CL/F (CL_tMPA_/F) of Asians and Caucasians was close (POT ≤ 6 months: 0.35 *vs.* 0.36 L/h/kg; POT > 6 months: 0.22 *vs.* 0.25 L/h/kg) following coadministration of CsA (Li et al., 2014). In contrast, several studies showed that MPA PK in Asians was similar to that in Caucasians (Funaki, 1999; Cho et al., 2004; Jiao et al., 2007; Ling et al., 2015). In addition, our previous study suggested that the lower dose required in Chinese patients than in Caucasians could result in comparable inhibitory rates of inosine-5′-monophosphate dehydrogenase (24–42%) (Liu et al., 2018). The difference in dose requirement might not be attributable to PK but to pharmacodynamics.

The current study showed no obvious ethnic difference in exposure of uMPA. As shown in **Table 3**, the population estimate of BW corrected-CL_uMPA_/F (13.95 L/h/kg) was comparable to most of previously reported values in Caucasians (9.21–12.93 L/h/kg) (de Winter et al., 2009; van Hest et al., 2009; Colom et al., 2018). Moreover, the typical value of BW corrected-CL_tMPA_/F (0.26 L/h/kg) was also similar to most of previously reported values in Asians (0.21–0.32 L/h/kg) (Yau et al., 2009; Yu et al., 2017; Chen et al., 2019) and Caucasians (0.18–0.32 L/h/kg) (Le Guellec et al., 2004; Cremers et al., 2005; Staatz et al., 2005; van Hest et al., 2007; de Winter et al., 2008; Musuamba et al., 2009; Guillet et al., 2010; de Winter et al., 2012; Colom et al., 2014). Furthermore, the population estimate of FU_MPA_ in our study (1.84%) was also similar to previously reported values (van Hest et al., 2009; Colom et al., 2018).

**Table 3:** Previously published population pharmacokinetic analysis of unbound and total mycophenolic acid.

References	Present study	Okour et al., 2018	Colom et al., 2018	van Hest et al., 2009	de Winter et al., 2009
Number of patients	58	92	56	88	75
Ethnicity	Chinese (100%)	Caucasian (93%)	Caucasian (majority)	Caucasian (95%)	Caucasian (majority)
Body weight, kg	61 (40.5–82.5)	82.3 (/)	71 (35–100)	67 (40–99)	67 (42–99)
Concomitant CNI	CsA	CsA/TAC	CsA/TAC	CsA	CsA/TAC
Postoperative time	3-3084 days	/	7 days–1 year	7–148 days	4–155 days
Structure model	MPA: 2 CMT MPAG: 1 CMT	MPA: 1 CMT MPAG: 1 CMT	MPA: 2 CMT	MPA: 2 CMT MPAG: 2 CMT	MPA: 2 CMT MPAG: 1 CMT
*pharmacokinetic parameter^a^*					
FU_MPA_, %	1.84 (2.3%)^b^	2.4 (5.2%)	1.93 (3.13%)^b^	2.03 (3%)^b^	/
CL_uMPA_/F, L/h	851 (7.1%)	1,832 (6.5%)	654 (3%)	866 (6%)	747 (/)
V_CuMPA_/F, L	718 (18.5%)	5,630 (7.9%)	18.3 (19.18%)	2,990 (27%)	189 (/)
Q_uMPA_/F, L/h	857 (11.0%)	/	749 (3.14%)	1,210 (13%)	2,010 (/)
V_PuMPA_/F, L	34,300 FIXED (/)	/	29,100 (8.59%)	6,240 (26%)	34,300 (/)
*Between-subject variability, %CV*					
CL_uMPA_/F	51.0 (11.0%)	30.1 (25.4%)	26.81 (69.82%)	25 (32%)	97 (/)
V_CuMPA_/F	80.0 (25.2%)	35.5 (33.6%)	99.45 (36.91%)	91 (30%)	116 (/)
*Between-occasion variability, %CV*					
CL_uMPA_/F	/	/	40.9 (52.1%)	/	/
V_CuMPA_/F	/	/	137.6 (22%)	/	/
*Residual unexplained variability, %CV*					
uMPA	47.0 (3.5%)	40.5 (9%)	58.3 (47.35%)	44 (6%)	99.3 (/)
tMPA	45.9 (3.7%)	35.8 (10.9%)	46.9 (4.18%)	42 (6%)	52 (/)

MPA, mycophenolic acid; MPAG, 7-O-mycophenolic acid glucuronide; tMPA, total MPA; uMPA, unbound MPA; /, not applicable or not available; %CV, percentage coefficient of variation; CL_uMPA_/F, apparent clearance of uMPA; CMT, compartment; CNI, calcineurin inhibitor; CsA, cyclosporine; FU_MPA_, unbound fraction of MPA; Q_uMPA_/F, apparent intercompartmental clearance of uMPA; TAC, tacrolimus; V_CuMPA_/F, apparent central volume of distribution of uMPA; V_PuMPA_/F, apparent peripheral volume of distribution of uMPA.

a Represented as typical values (relative standard error, RSE) for reference subjects: 1) body weight 70 kg, 2) serum albumin concentration 40 g/L, 3) glomerular filtration rate 90 mL/min, 4) cotreated with CsA 300 mg per day, 5) total MPAG concentration 0.1 mmol/L.

b Calculated based on the protein binding rate constant.

The stepwise covariate analyses suggested that ALB had significant effects on *k*_B_ and FU_MPA_. MPA is extensively bound to human ALB, which has more than one binding site on each molecule with equivalent binding characteristics (Nowak and Shaw, 1995). A reduction in ALB decreases the binding sites, which increases the FU_MPA_. The simulations showed that changes in ALB concentrations substantially affected FU_MPA_ and tMPA exposure, but had little effect on uMPA exposure, which was consistent with previous findings (de Winter et al., 2009; van Hest et al., 2009). This could be attributed to the low hepatic extraction ratio of MPA. FU_MPA_ tended to increase with decreasing ALB (**Supplementary Figure 1**) and the increase in FU_MPA_ caused relatively more uMPA to be metabolized and eliminated from the body, thereby decreasing tMPA exposure. In contrast, MPA is characterized by a low hepatic extraction ratio of 0.2 (Bowalgaha and Miners, 2001), and the unbound exposure of drugs with low extraction ratio is unaffected by changes in the unbound fraction (Benet and Hoener, 2002).

These results suggested that dose adjustment based on tMPA exposure might not be appropriate under lower ALB conditions. A reduction in median tMPA AUC_0–12h_ from approximately 42.9 to 21.6 mg·h/L was observed when ALB concentration decreased from 40 to 20 g/L for patients administered 750 mg MMF every 12 h. However, this observation does not indicate an MMF dose increment is necessary because of the unchanged uMPA exposure. Although a relationship between uMPA exposure and acute rejection risk has not been fully identified, uMPA has been recognized as the pharmacologically active component. Moreover, uMPA exposure has been demonstrated to be associated with the risk of leucopoenia and infections (Kaplan et al., 1998; Weber et al., 2002; Mudge et al., 2004; Atcheson et al., 2005). An increased MMF dose would also increase uMPA exposure, placing patients at a higher risk of overimmunosuppression with manifestations such as leucopoenia and infections. In such situations, monitoring uMPA exposure might be preferable to monitoring tMPA for adjusting the MMF dose.

Moreover, it has been reported that tMPA exposure in the early phase post-transplantation was 30–50% lower than that in the stable period when the same MMF dose was administered (Shaw et al., 2003). This time-dependent clearance could be largely attributed to changes in protein binding, resulted by increasing GFR, ALB, and hemoglobin levels with extension of time after transplantation (van Hest et al., 2007). In the present study, the influence of POT was reflected in corresponding changes in ALB concentrations, which significantly impacted FU_MPA_. Alterations of protein binding had little effect on uMPA PK because of the low extraction ratio (Bowalgaha and Miners, 2001; Benet and Hoener, 2002). In addition, the significant positive association between Q_uMPA_/F and BW was observed in the present study. This relationship is consistent with the known physiological properties.

Additionally, a gallbladder compartment was introduced to characterize the intermittent EHC process in the current popPK analysis. Generally, the intermittent gallbladder emptying process is considered to be triggered by ingestion of food (Ghibellini et al., 2006). The EHC process is mediated by multidrug resistance-associated protein 2, which is inhibited by CsA (Hesselink et al., 2005). All subjects in our study were cotreated with CsA. Meal time was set at 10 (study 1) and 9 (study 2) h postdosing, and the samplings before and after food intake were considered in the study protocol. The EHC triggered by food intake was applied during modeling. Nevertheless, the secondary peaks were not pronounced due to the inhibitory effect of CsA. Therefore, inhibition of EHC by CsA might likely explain why the final model estimated an extremely low %EHC with a high shrinkage (> 50%).

Regarding the metabolite MPAG, a statistically significant relationship was found between CL_uMPAG_/F and kidney function, which was consistent with findings of previous studies (de Winter et al., 2009; Musuamba et al., 2009; van Hest et al., 2009; Colom et al., 2014). A reduction in GFR from 90 to 15 mL/min led to a 3.67-fold increase in MPAG exposure. This could be because MPAG is primarily eliminated by the kidney through passive glomerular filtration and active tubular secretion (Bullingham et al., 1998).

Nevertheless, the previously reported competitive protein binding relationship between MPA and MPAG (de Winter et al., 2009; van Hest et al., 2009) was not observed, which might be associated with the relatively lower MPAG concentrations (median, 49.79 mg/L). Only 7.6% (56/734) of the tMPAG concentrations were >100 mg/L with a maximum of 177.9 mg/L in our study. At high concentrations, MPAG could displace MPA from its protein binding sites. It has been reported *in vitro* that FU_MPA_ increased threefold as the MPAG concentration increased from 0 to 800 mg/L (Nowak and Shaw, 1995).

There are some limitations in the present study. Firstly, uMPAG concentrations were not determined and FU_MPAG_ was fixed at 18%. The effect of ALB alteration on MPAG binding could not be investigated. MPAG is pharmacologically inactive and did not show significant influence on MPA PK in our analysis because of the relatively lower MPAG concentrations. Secondly, only one dose level of MMF was administered to most patients, which prevented us from investigating the reported nonlinear relationship between MMF dose and MPA exposure (de Winter et al., 2011). Lastly, all patients in our study were coadministered MMF and CsA, whereas TAC and CsA are well known to influence the EHC process differently. Therefore, our results might only be applicable to patients cotreated with CsA.

## Conclusions

In summary, the established model adequately described the popPK characteristics of uMPA, tMPA, and MPAG. Large BSVs and RUVs were still observed, suggesting therapeutic drug monitoring would be necessary for optimization of MMF therapy. The estimated CL_uMPA_/F and FU_MPA_ in Chinese kidney transplant recipients were comparable to those published previously in Caucasians. In addition, tMPA exposure reduced with decreasing ALB, which had little effect on uMPA exposure. Therefore, under lower ALB conditions, dose adjustment based on tMPA exposure might place patients at higher risk of overimmunosuppression. We recommend monitoring uMPA instead of tMPA to optimize MMF dosing for patients with lower ALB concentrations.

## Data Availability Statement

The datasets for this article are not publicly available because of privacy and ethical restrictions. Requests to access the datasets should be directed to corresponding author (ZJ, zjiao@fudan.edu.cn).

## Ethics Statement

The studies involving human participants were reviewed and approved by the Clinical Research Ethics Committee of Huashan Hospital, Fudan University. The patients/participants provided their written informed consent to participate in this study.

## Author Contributions

ZJ, XQ, and MZ participated in the research design. CS, QZ, WN, XQ, and MZ contributed to the implementation of the research. ZJ, CS, QZ, and WN analyzed and interpreted the data. CS and ZJ drafted the manuscript. The final submitted version of the manuscript was reviewed and approved by all the authors.

## Funding

This work was supported by the National Natural Science Foundation of China (No. 81573505), Weak Discipline Construction Project of Shanghai Municipal Commission of Health and Family Planning (No. 2016ZB0301-01), and 2016 Key Clinical Program of Clinical pharmacy of Shanghai Municipal Commission of Health and Family Planning.

## Conflict of Interest

The authors declare that the research was conducted in the absence of any commercial or financial relationships that could be construed as a potential conflict of interest.

## Supplementary Material

Supplementary Table 1 Key covariate model development steps

Supplementary Table 2 Sensitivity analysis of observations over ±4 CWRES

Supplementary Figure 1 Model-predicted covariate effect on unbound fraction of MPA. Black squares represent median values and error bars represent 95% confidence intervals of unbound fraction of MPA across 2000 simulation replicates. GFR, glomerular filtration rate; MPA, mycophenolic acid.
