# Supplementary material for: Effect of Protein Binding on Exposure of Unbound and Total Mycophenolic Acid: A Population Pharmacokinetic Analysis in Chinese Adult Kidney Transplant Recipients
Source: Front Pharmacol. 2020 Mar 20;11:340. doi: 10.3389/fphar.2020.00340 (PMC7100081; doi:10.3389/fphar.2020.00340)
Supplement: Supplementary Table 1 — Key covariate model development steps [file DataSheet_1.docx]

Supplementary Material

# Supplementary Figures and Tables

## Supplementary Tables

### Supplementary Table 1. Key covariate model development steps

| **Model No.** | **Model description** | **OFV** | **ΔOFV** | **Significance ^a^** | ***df*** | **Screening results** | **Comparator** |
| --- | --- | --- | --- | --- | --- | --- | --- |
| 001 | Base model | 2902.028 | / | / | / | / | / |
| ***Forward inclusion step 1*** | |  |  |  |  |  |  |
| 101 | Add BW on CL_uMPA_/F | 2897.188 | -4.84 | Yes | 1 | / | 001 |
| 102 | Add BW on Q_uMPA_/F | 2875.843 | -26.185 | **Yes** | 1 | / | 001 |
| 103 | Add BW on V_CuMPA_/F | 2897.418 | -4.61 | **Yes** | 1 | / | 001 |
| 104 | Add BW on CL_uMPAG_/F | 2895.670 | -6.358 | Yes | 1 | / | 001 |
| 105 | Add BW on V_CuMPAG_/F | 2898.348 | -3.68 | No | 1 | / | 001 |
| 106 | Add GFR on *k*_B_ | 2887.736 | -14.292 | **Yes** | 1 | / | 001 |
| 107 | Add GFR on CL_uMPA_/F | 2900.309 | -1.719 | No | 1 | / | 001 |
| 108 | Add GFR on Q_uMPA_/F | 2899.334 | -2.694 | No | 1 | / | 001 |
| 109 | Add GFR on CL_uMPAG_/F | 2860.891 | -41.137 | **Yes** | 1 | / | 001 |
| 110 | Add ALB on *k*_B_ | 2825.253 | -76.775 | **Yes** | 1 | Included | 001 |
| 111 | Add ALB on V_CuMPA_/F | 2882.637 | -19.391 | **Yes** | 1 | / | 001 |
| 112 | Add ALB on V_CuMPAG_/F | 2889.373 | -12.655 | **Yes** | 1 | / | 001 |
| 113 | Add ANTAC on CL_uMPA_/F | 2898.843 | -3.185 | No | 1 | / | 001 |
| 114 | Add ANTAC on *k*_a_ | 2893.362 | -8.666 | **Yes** | 1 | / | 001 |
| 115 | Add SEX on CL_uMPA_/F | 2890.549 | -11.479 | **Yes** | 1 | / | 001 |
| 116 | Add SEX on Q_uMPA_/F | 2875.957 | -26.071 | **Yes** | 1 | / | 001 |
| 117 | Add SEX on V_CuMPA_/F | 2898.940 | -3.088 | No | 1 | / | 001 |
| 118 | Add SEX on CL_uMPAG_/F | 2900.461 | -1.567 | No | 1 | / | 001 |
| 119 | Add SEX on V_CuMPAG_/F | 2897.371 | -4.657 | **Yes** | 1 | / | 001 |
| 120 | Add MPAG concentration on *k*_B_ | 2899.468 | -2.56 | No | 1 | / | 001 |
| 121 | Add MPAG trough concentration on *k*_B_ | 2898.254 | -3.744 | No | 1 | / | 001 |
| ***Forward inclusion step 2*** | |  |  |  |  |  |  |
| 201 | Add BW on CL_uMPA_/F | 2825.341 | 0.088 | No | 1 | / | 110 |
| 202 | Add BW on Q_uMPA_/F | 2796.479 | -28.774 | **Yes** | 1 | / | 110 |
| 203 | Add BW on V_CuMPA_/F | 2826.504 | 1.251 | No | 1 | / | 110 |
| 204 | Add BW on CL_uMPAG_/F | 2823.186 | -2.067 | No | 1 | / | 110 |
| 205 | Add GFR on *k*_B_ | 2828.097 | 2.844 | No | 1 | / | 110 |
| 206 | Add GFR on CL_uMPAG_/F | 2786.030 | -39.223 | **Yes** | 1 | Included | 110 |
| 207 | Add ALB on V_CuMPA_/F | 2811.199 | -14.054 | **Yes** | 1 | / | 110 |
| 208 | Add ALB on V_CuMPAG_/F | 2824.181 | -1.072 | No | 1 | / | 110 |
| 209 | Add ANTAC on *k*_a_ | 2820.533 | -4.72 | **Yes** | 1 | / | 110 |
| 210 | Add SEX on CL_uMPA_/F | 2826.242 | 0.989 | No | 1 | / | 110 |
| 211 | Add SEX on Q_uMPA_/F | 2806.506 | -18.747 | **Yes** | 1 | / | 110 |
| 212 | Add SEX on V_CuMPAG_/F | 2833.594 | 8.341 | No | 1 | / | 110 |
| ***Forward inclusion step 3*** | |  |  |  |  |  |  |
| 301 | Add BW on Q_uMPA_/F | 2760.054 | -25.976 | **Yes** | 1 | Included | 206 |
| 302 | Add ALB on V_CuMPA_/F | 2771.657 | -14.373 | **Yes** | 1 | / | 206 |
| 303 | Add ANTAC on *k*_a_ | 2779.509 | -6.521 | **Yes** | 1 | / | 206 |
| 304 | Add SEX on Q_uMPA_/F | 2777.595 | -8.435 | **Yes** | 1 | / | 206 |
| ***Forward inclusion step 4*** | |  |  |  |  |  |  |
| 401 | Add ALB on V_CuMPA_/F | 2746.482 | -13.572 | **Yes** | 1 | Not included ^b^ | 301 |
| 402 | Add ANTAC on *k*_a_ | 2753.658 | -6.396 | **Yes** | 1 | / | 301 |
| 403 | Add SEX on Q_uMPA_/F | 2751.028 | -9.026 | **Yes** | 1 | Included | 301 |
| ***Forward inclusion step 5*** | |  |  |  |  |  |  |
| 501 | Add ALB on V_CuMPA_/F | 2736.498 | -14.53 | **Yes** | 1 | Not included ^b^ | 403 |
| 502 | Add ANTAC on *k*_a_ | 2749.276 | -1.752 | No | 1 | / | 403 |
| ***Backward elimination step 1*** | |  |  |  |  |  |  |
| 601 | Eliminate ALB from *k*_B_ | 2828.610 | 77.582 | **Yes** | 1 | Retained | 403 |
| 602 | Eliminate GFR from CL_uMPAG_/F | 2788.455 | 37.427 | **Yes** | 1 | Retained | 403 |
| 603 | Eliminate BW from Q_uMPA_/F | 2777.595 | 26.567 | **Yes** | 1 | Retained | 403 |
| 604 | Eliminate SEX from Q_uMPA_/F | 2760.054 | 9.026 | **No** | 1 | Eliminated | 403 |
| ***Backward elimination step 2*** | |  |  |  |  |  |  |
| 701 | Eliminate ALB from *k*_B_ | 2833.893 | 73.839 | **Yes** | 1 | Retained | 604 |
| 702 | Eliminate GFR from CL_uMPAG_/F | 2796.479 | 36.245 | **Yes** | 1 | Retained | 604 |
| 703 | Eliminate BW from Q_uMPA_/F | 2786.030 | 25.976 | **Yes** | 1 | Retained | 604 |

MPAG, 7-O-mycophenolic acid glucuronide; uMPA, unbound mycophenolic acid; uMPAG, unbound MPAG; ALB, serum albumin; ANTAC, antacid; BW, body weight; CL_uMPA_/F and CL_uMPAG_/F, apparent clearance of uMPA and uMPAG, respectively; *df*, degree of freedom; GFR, glomerular filtration rate; *k*_a_, absorption rate constant; *k*_B_, protein binding rate constant; OFV, objective function value; Q_uMPA_/F, apparent intercompartmental clearance of uMPA; V_CuMPA_/F and V_CuMPAG_/F, apparent central volume of distribution of uMPA and uMPAG, respectively; ΔOFV, change in OFV

^a^ Significance levels are set at a decrease in OFV of >3.84 (χ^2^, *df* =1, *p* <0.05) during the forward inclusion step and an increase in OFV of >10.83 (χ^2^, *df* =1, *p* <0.001) during the backward elimination step, respectively.

^b^ Addition of ALB on V_CuMPA_ results in a significant decrease in OFV. However, it also leads to an increase in the between-subject variability on V_CuMPA_ of over 55%. Thus, ALB is not included as a significant covariate.

### Supplementary Table 2. Sensitivity analysis of observations over ±4 CWRES

| **Parameters** | **Model 901**  **(Final model)** | **Model 901.2**  **(Exclusion of observations**  **over ±4 CWRES)** | **Relative bias (%)** |
| --- | --- | --- | --- |
| *Pharmacokinetic parameters* |  |  |  |
| CL_uMPA_/F, L/h | 851 | 863 | 1.41 |
| Q_uMPA_/F, L/h | 857 | 867 | 1.17 |
| Exponent for the effect of BW on Q_uMPA_/F | 2.11 | 1.98 | -6.16 |
| V_CuMPA_/F, L | 718 | 733 | 2.09 |
| *k*_a_ , /h | 1.35 | 1.33 | -1.48 |
| Tlag ,h | 0.447 | 0.438 | -2.01 |
| *k*_B_, /h | 53.4 | 53.1 | -0.56 |
| CL_uMPAG_/F, L/h | 5.71 | 5.72 | 0.18 |
| Exponent for the effect of GFR on CL_uMPAG_/F | 0.865 | 0.844 | -2.43 |
| V_CuMPAG_/F, L | 29.9 | 29.6 | -1.00 |
| %EHC | 5.53 | 5.60 | 1.27 |
| *Between-subject variability, %CV* |  |  |  |
| CL_uMPA_/F | 51.0 | 47.6 | -6.67 |
| Q_uMPA_/F | 45.5 | 44.8 | -1.54 |
| V_CuMPA_/F | 80.0 | 78.8 | -1.50 |
| *k*_a_ | 46.5 | 43.4 | -6.67 |
| Tlag | 107.7 | 108.6 | 0.84 |
| CL_uMPAG_/F | 31.8 | 31.0 | -2.52 |
| Correlation between CL_uMPAG_/F and V_CuMPAG_/F | 57.4 | 57.3 | -0.17 |
| V_CuMPAG_/F | 48.4 | 49.0 | 1.24 |
| %EHC | 61.6 | 61.5 | -0.16 |
| *Residual unexplained variability, %CV* |  |  |  |
| uMPA | 47.0 | 46.6 | -0.85 |
| Correlation between uMPA and tMPA | 51.2 | 48.8 | -4.69 |
| tMPA | 45.9 | 45.1 | -1.74 |
| uMPAG | 22.0 | 22.0 | 0.00 |

MPA, mycophenolic acid; MPAG, 7-O-mycophenolic acid glucuronide; tMPA, total MPA; uMPA, unbound MPA; uMPAG, unbound MPAG; %CV, percentage coefficient of variation; %EHC, percentage of MPAG recycled into the systemic circulation; BW, body weight; CL_uMPA_/F and CL_uMPAG_/F, apparent clearance of uMPA and uMPAG, respectively; CWRES, conditional weighted residuals; GFR, glomerular filtration rate; *k*_a_, absorption rate constant; *k*_B_, protein binding rate constant; Q_uMPA_/F, apparent intercompartmental clearance of uMPA; Tlag, lagged absorption time; V_CuMPA_/F and V_CuMPAG_/F, apparent central volume of distribution of uMPA and uMPAG, respectively

## Supplementary Figures


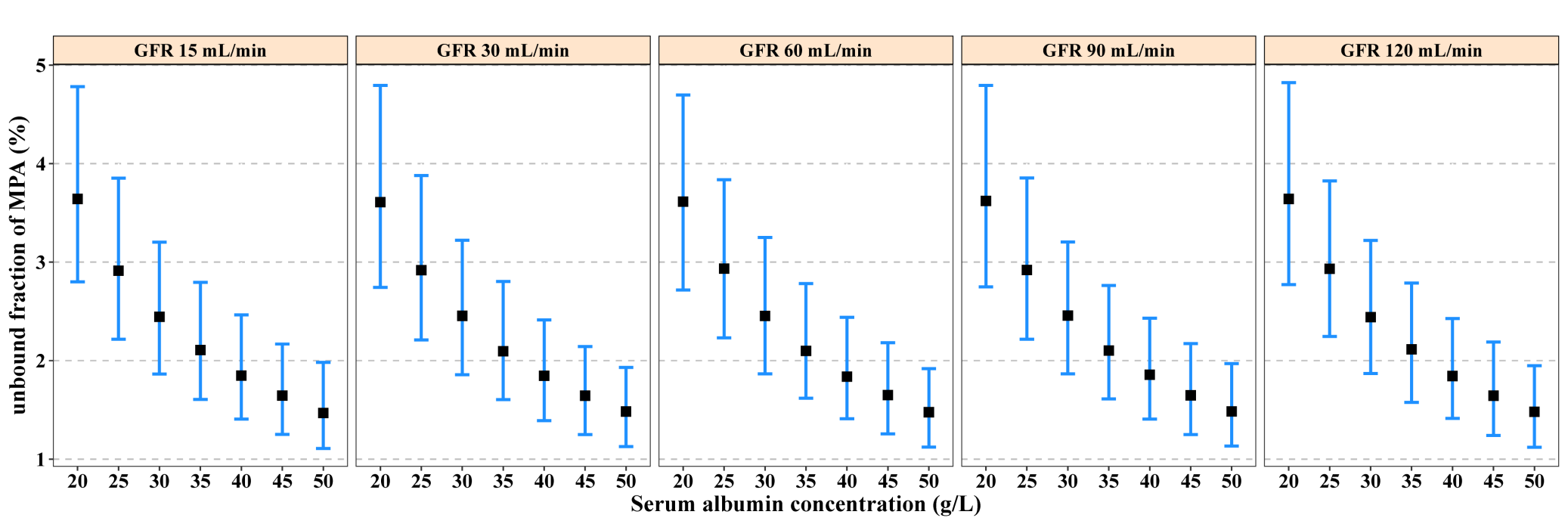


### **Supplementary Figure 1.** Model-predicted covariate effect on unbound fraction of MPA. Black squares represent median values and error bars represent 95% confidence intervals of unbound fraction of MPA across 2000 simulation replicates. GFR, glomerular filtration rate; MPA, mycophenolic acid
